# BISHOP-KOOP OSTOMY REVISITED: A “TEST-DRIVE” INTESTINAL DIVERSION FOR CHILDREN WITH SUSPECTED BOWEL DYSMOTILITY

**DOI:** 10.1590/0102-672020230002e1722

**Published:** 2023-03-20

**Authors:** Márcia Alessandra Cavalaro Pereira da Silva, Márcio Lopes Miranda, António Gonçalves Oliveira-Filho, Joaquim Murray Bustorff-Silva

**Affiliations:** 1 Universidade Estadual de Campinas, Faculty of Medical Sciences, Department of Surgery, Pediatric Surgery Division – Campinas (SP), Brazil.

**Keywords:** Ileostomy. Gastrointestinal Motility, Child, Meconium Ileus, Ileostomia, Motilidade gastrointestinal, Criança, Íleo meconial

## Abstract

**BACKGROUND:**

Bishop-Koop ileostomy has been widely used in pediatric patients with the intention of including as much bowel as possible in the intestinal transit early in the management of children with meconium ileus and intestinal atresia. In recent years, we have been using it as an alternative to test the distal bowel function before closure of a previously constructed ostomy in selected children with questionable distal bowel motility.

**AIMS:**

The aim of this study was to present our experience with this alternative use of the Bishop-Koop ostomy.

**METHODS:**

This is a cross-sectional retrospective review of hospital records, combined with a comprehensive literature review.

**RESULTS:**

Seven children were included: five had suspected aganglionosis, one had gastroschisis complicated with ileal atresia, and one had a colonic stricture secondary to necrotizing enterocolitis. In this short series of patients, motility of the distal bowel was correctly assessed in six patients and partially correctly assessed in one patient. One patient did not pass stools *per anus* after the Bishop-Koop, and he was later confirmed to have Hirschsprung disease. Four patients resumed normal evacuation pattern after closure of the Bishop-Koop. One patient had a Bishop-Koop colostomy because of recurrent enterocolitis after a transanal pull-through. Although he evacuated normally while having the colostomy, the diarrhea recurred after the ostomy was closed. An additional patient, with a severe behavioral problem, did not evacuate *per anus* after her colostomy was transformed in a Bishop-Koop-type ostomy, despite the apparent presence of normal ganglia in the bowel wall.

**CONCLUSIONS:**

Data from the present series allow us to affirm that Bishop-Koop-type ostomy is a safe and efficient procedure that can be used to assess distal bowel function before a definitive transit reconstruction, in children with uncertain motility issues.

## RESUMO

**RACIONAL:**

A ileostomia Bishop-Koop foi amplamente utilizada em pacientes pediátricos com a intenção de incluir o máximo de intestino possível no trânsito intestinal no manejo inicial de recém-nascidos com íleo meconial e atresia intestinal. Nos últimos anos, temos usado-a como alternativa para testar a função intestinal distal antes do fechamento de uma ostomia, em algumas crianças com motilidade intestinal distal questionável.

**OBJETIVOS:**

Apresentar nossa experiência com este uso alternativo da ostomia Bishop-Koop.

**MÉTODOS:**

Revisão retrospectiva dos registros hospitalares, combinada com uma revisão abrangente da literatura.

**RESULTADOS:**

Sete crianças foram incluídas: cinco tinham suspeita de aganglionose, uma tinha gastrosquise complicada com atresia ileal e uma tinha estenose de colon secundária à NEC. Nesta pequena série de pacientes, a motilidade do intestino distal foi corretamente avaliada em 6 pacientes e parcialmente avaliada em um. Um paciente não evacuou por ânus após o Bishop-Koop e mais tarde foi confirmado que ele tinha doença de Hirschsprung. Seis pacientes retomaram o padrão normal de evacuação após o fechamento do Bishop-Koop. Um paciente que fez uma colostomia Bishop-Koop por causa de enterocolite recorrente após um abaixamento transanal, recidivou a enterocolite após o fechamento definitivo.

**CONCLUSÕES:**

A ostomia tipo Bishop-Koop é um procedimento seguro e eficaz que pode ser utilizado para avaliar a função intestinal distal antes de uma reconstrução definitiva do trânsito em crianças com problemas de motilidade intestinal.

## INTRODUCTION

Many children are referred to a tertiary hospital, after having a diverting ostomy placed as an urgent operation, because of an intestinal obstruction or perforation of unidentified cause, at a local hospital, sometimes by an unprepared surgeon.

Once in the tertiary hospital, these children usually undergo a series of diagnostic and functional tests in order to establish the underlying diagnosis and ascertain distal bowel integrity. Although this work-up is mostly successful, in a small number of children, because of inconsistency among the results of the different diagnostic tests, variations in pathological criteria of enteric dysganglionosis, or the eventual lack of an experienced pediatric pathologist, a definitive etiologic and functional diagnosis may be very difficult to establish.

In these situations, attempting to close the enterostomy without being absolutely certain of the normal function of the distal bowel may lead to an unacceptable rate of anastomotic disruption^19,21^.

Bishop-Koop (BK) ileostomy is a technique designed by Bishop et al. in 1957, specifically to treat children with “complicated meconium ileus”^4^. Due to its simplicity, this technique has been employed to treat other conditions other than meconium ileus (MI), such as jejunoileal atresia^23,24^, neonatal necrotizing enterocolitis (NEC)^14^, and other types of neonatal intestinal obstruction, allowing for early integration of the distal bowel in the intestinal transit^1^.

Recently, we have been using it as an alternative to test the distal bowel function before closure of a previously constructed ostomy in selected children in whom doubts regarding distal intestinal motility could not be clarified and primary reconstruction was considered unsafe. The aim of this study was to present our experience with this alternative use of the BK ostomy.

## Methods

### Patients

The present study was approved by the National Ethic Committee on research involving human subjects (CAAE nº 49321021.6.0000.5404).

In the past 8 years, seven children received a BK-type ostomy at the University Hospital of Faculty of Medicine, Universidade Estadual de Campinas – Unicamp, due to uncertain distal bowel dysmotility, which forms the basis of this report (Table 1). There were two males and five females. Age at BK operation varied between 9 days and 38 months (19.5±12 months). Underlying diagnoses included suspected aganglionosis of varied extension in five (one associated with Down syndrome), one with an intestinal stricture secondary to neonatal NEC, and one with gastroschisis associated with ileal atresia. The reasons for performing a BK ostomy were as follows: inconsistent pathology results in six children (four with aganglionosis, one with post-NEC stricture, and one with ileal atresia associated with gastroschisis) and recurring enterocolitis in the child with Down syndrome and intestinal aganglionosis. All the children were operated on an elective basis, after a thorough clinical-pathological case discussion by the whole pediatric surgery team. Decision to proceed with a temporary BK was taken if the following criteria were met: the child had been admitted for closure of a previous diverting stoma and, despite extensive radiological, manometric, and pathological evaluation, motility of the distal colon remained doubtful, and primary closure was considered unsafe. This decision was discussed with the families, and surgery was scheduled only after family agreement.

**Table 1:** Clinical data of the five patients who received a Bishop-Koop ostomy in the past 8 years.

	Gender	Diagnosis	Initial operation	Number of operations	Age at BK (months)	Indication of BK	Evacuations post BK	Outcome
1	M	Aganglionosis	Double barrel ileostomy	1	17	Inconclusive diagnostic work-up	Ileal evacuations Duhamel pull-through	Good NEP
2	M	Hirschsprung + Down syndrome	Colostomy	3	26	Post pull-through constipation	Anal evacuations colostomy closure	Recurrent diarrhea
3	F	Gastroschisis + ileal atresia	Double barrel ileostomy	5	22	Uncertain bowel motility	Anal evacuations ileostomy closure	Good NEP
4	F	Post NEC stenosis	Colostomy	2	38	Inconclusive diagnostic work-up	Anal evacuations colostomy closure	Good NEP
5	F	Aganglionosis	Double barrel ileostomy	1	13	Inconclusive diagnostic work-up	Anal evacuations ileostomy closure	Good NEP
6	F	Hypoganglionosis	Double barrel ileostomy	1	13	Inconclusive diagnostic work-up	Anal evacuations ileostomy closure	Good NEP
7	F	Aganglionosis	Colostomy	1	38	Inconclusive diagnostic work-up	Partial evacuation per anus	Waiting to close the colostomy

NEP: normal evacuation pattern; BK: Bishop-Koop ileostomy; NEC: neonatal necrotizing enterocolitis.

### Surgical technique

**Figure 1:**
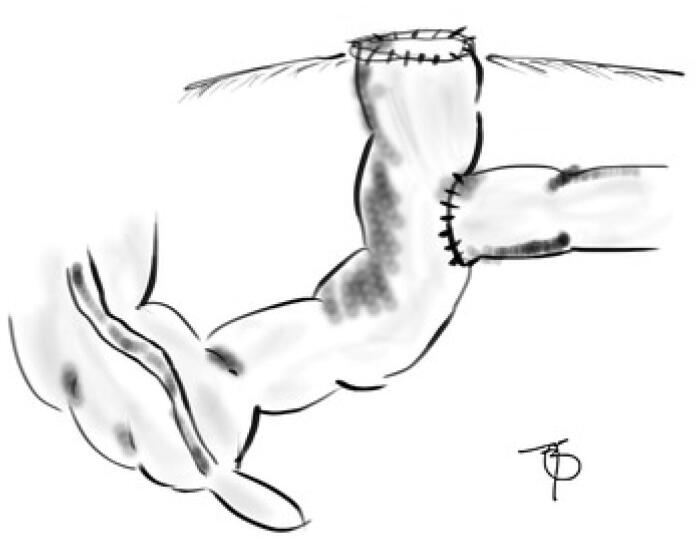
The Bishop-Koop-type ostomy. The efferent limb is attached to the skin to serve as a vent to decompress the distal bowel in case of persisting dysmotility.

The BK ostomy was performed in the operation theater under general anesthesia in all children included in this report. All the procedures were performed by a resident fellow assisted by one of the four staff pediatric surgeons. Upon abdominal exploration, appropriate surgical interventions were performed to solve the initial problem. Then, the ostomy was initiated by making an incision in the distal intestinal antimesenteric wall large enough to accommodate the diameter of the proximal intestine. End-side anastomosis of the proximal and distal bowels was performed, resulting in a T-shaped anastomosis. The proximal end of the distal intestine was pulled outside the skin incision as a single-lumen enterostomy with a preserved length of 2 cm. An inverted stoma was constructed, and the seromuscular layer of the intestinal wall was secured to the peritoneum and muscle sheath using an absorbable suture. The cavity was closed in layers, as usual (Figure 1).

## RESULTS

Postoperative course was uneventful in all children. Four children presented anal evacuations 2–4 days after surgery and had normal evacuations after subsequent closure of the BK (Table 1). The child with associated aganglionosis and Down syndrome, who had a colostomy placed because of recurrent enterocolitis after initial pull-through, took 40 days to evacuate per anus once the colostomy was converted into a BK. As the intraoperative biopsies showed normal intestinal ganglia, the BK was eventually closed, but the child still presents recurrent bouts of mild-to-moderate enterocolitis. Another child, with suspected aganglionosis, but who had a pathology report of “reduced number of intestinal neurons,” never passed stools *per anus* during the period he had a BK. A later laparotomy revealed a classic transition zone, confirmed by intraoperative biopsies, and he was treated successfully by a Duhamel pull-through. An additional patient, with a severe behavioral problem and an unconfirmed previous diagnosis of hypoganglionosis, did not evacuate *per anus* after her colostomy was transformed in a BK-type ostomy, despite the fact that histological revision showed the presence of normal ganglia in the bowel wall. This latter child is presently under psychiatric care and waiting to close her BK colostomy. Overall, the BK correctly assessed the patency of the distal bowel in all children, allowing for the correct choice of definitive treatment.

## DISCUSSION

In infants, ostomy opening and closing are often the required steps to treat congenital conditions such as anorectal malformations and Hirschsprung disease (HD), or acquired conditions such as NEC. Several types of intestinal stomas have been described for use in children: end stomas, double-barrel stomas, Nixon's skin bridge stoma, loop stoma, BK, and Santulli stomas^1,25^.

Although closure of an ostomy is a common and straightforward procedure, it is not free of complications, and the incidence of anastomotic leaks is reported to be between 1.6 and 31% in children with an increased risk of anastomotic complications, such as peritonitis (NEC) or a size discrepancy between the dilated proximal and the unused distal bowel (MI). In these cases, a two-stage restoration of intestinal continuity using the formation of the diverting enterostomy has been advocated^2,13,15,20,31^.

The BK ileostomy was initially designed for the treatment of infants with MI^4^. It reduces intestinal fluid losses and is easily reversible. When compared to a divided stoma, the BK has less stoma-related complications (8.7 vs. 31%), less complications after reversal (6.7 vs. 3.5%), and shorter operating time and length of hospital stay for ostomy reversal. In cases of intestinal transit problems, the “chimney” could act as a safety vent, partially decompressing the anastomosis and reducing the risk of anastomotic breakdown. Due to its advantages, it has gradually been adapted for many other purposes, such as jejunoileal atresia, NEC, short bowel syndromes, and even intestinal transplantation^5,21,23,24,26^.

These characteristics make this type of intestinal diversion ideal to use as a “test-drive” in children referred for ostomy closure in whom normal intestinal motility cannot be safely confirmed.

Despite the different underlying diagnosis, all children in this series share a background of conflicting results regarding motility and integrity of the myenteric plexus. One with neonatal clinical evidence of HD had an initial intestinal biopsy, showing “reduced number of myenteric neurons.” This child never evacuated *per anus* after the BK. A later laparotomy confirmed the diagnosis of HD, and the child was treated with the Duhamel procedure. Another child, who had an ileostomy and was referred because of a pathological diagnosis of total colonic aganglionosis, had a revision of the initial biopsy, which showed a normal myenteric plexus. BK was performed because of the radiological finding of a very narrow distal colon. In the postoperative period, she evacuated normally after 2 days, allowing for definitive closure of the ostomy. Another child was a boy with Down syndrome with recurring enterocolitis after an endoanal pull-through. Doubts as to whether the recurrent enterocolitis was a result of poor motility, despite a biopsy showing the presence of neurons in the rectal and distal colon myenteric plexus, prompted the placement of the BK. He did well with the BK and underwent definitive closure after 1 year. The fact that this child continues to have enterocolitis even after the BK closure may be taken as indirect evidence that, eventually, normal histology does not equal normal motility^3,8,16^. The remaining child was referred to us with a suspected diagnosis of hypoganglionosis together with a clinical picture suggestive of HD. Histological revision showed normal myenteric plexuses in all segments of the bowel. As the child and the family presented severe behavioral disturbances, it was decided to transform her colostomy into a BK-type ostomy before definitive closure. As suspected, despite the normal bowel histology, the child did not evacuate *per anus* and is presently under psychological care and waiting to close her BK colostomy. It is believed that in this child, simple closure of the colostomy would be associated with a high risk of anastomotic rupture.

A literature-based systematic revision disclosed a mean sensitivity of rectal suction biopsy (RSB) of 96.84% and a mean specificity of 99.42% for RSB with acetylcholinesterase staining^11^ and approximately the same with calretinin immunostaining^7,22^. However, immunostaining techniques may be difficult to execute and may not be available as routine staining in every pathology laboratory.

An additional difficulty in assessing bowel motility based on the results of an intestinal biopsy is the presence of the so-called HD allied disorders. According to Friedmacher et al., “variants of Hirschsprung's disease” are conditions that clinically resemble HD, despite the presence of ganglion cells in rectal suction biopsies^10^. Criteria for classifying the different variants are continuously evolving, and new forms of intestinal dysganglionosis are constantly being described, bringing more confusion and doubt to an already bewildering field^17^. As a consequence, the common aphorism that “if a ganglion cell is found, then the child has not Hirschsprung's disease” does not guarantee that the bowel has normal motility^3,8,16^. Patients 1 and 3 had questionable diagnoses of hypoganglionosis, raising the concern that a variant of HD might be present.

Similar to what was found with rectal biopsies, contrast enema (CE) and anorectal manometry (ARM), when performed adequately, also display high sensitivity and specificity to diagnose HD^9,32^. However, most of the children with suspected HD or allied disorders undergo more than one diagnostic test, and conflicting results may occur, creating diagnostic and functional uncertainties. The presence of a diverting ostomy poses an additional challenge, because it may significantly alter the results of both CE and ARM^6,12^. Two of the children (4 and 5) in this series displayed discordant results between the CE and the biopsy findings.

Another clinical situation where biopsies can be misleading and the BK procedure can be handful is in children with a long-standing ostomy secondary to a NEC episode in early infancy as it happened in patient 4 of the present series. Some of these children may develop late intestinal strictures and ultimately need intestinal resection^18^. There are evidences that, even in milder forms of NEC, injury to the myenteric plexus may occur and cause motility problems^29,30^.

In these situations, pathology may be limited because of overlapping of histological findings between HD, neuronal intestinal dysplasia, and myenteric plexus injuries of NEC^29^. Additionally, frozen sections obtained at the time of the definitive laparotomy may not be adequate to diagnose motility problems^28^, supporting the creation of an interval BK instead of primary closure. These considerations may also apply in a situation of gastroschisis as in patient 3, as it is well known that patients with gastroschisis may have chronic motility disorders secondary to immaturity of the myenteric plexus that may involve different segments of the bowel^27^.

Therefore, although most of the times the integrity and function of the distal bowel can be safely assessed by the abovementioned methods, in the rare instance where, despite extensive diagnostic and functional work-up, motility and function of the distal bowel remain obscure, it is believed that constructing a BK-type ostomy may be a safe alternative procedure to “test-drive” the distal bowel and correctly assess its motility and function. If, after constructing the ostomy, the child begins to evacuate 100% per anus, then the transit can be reconstructed with confidence. Otherwise, further diagnostic workup might be warranted, or, if indicated, a pull-through operation might be scheduled to restore intestinal transit.

In this short series of patients, the motility of the distal bowel was correctly assessed in six patients and partially correctly assessed in one. This latter patient had HD associated with Down syndrome and had a colostomy constructed because of recurrent enterocolitis persisting after a transanal pull-through. Although he evacuated normally while having a BK colostomy in place, he had recurrent episodes of diarrhea after the ostomy was closed. It is of notice that, differently from the other children in this series, it took this child almost 2 months to start evacuating *per anus* once the BK was constructed.

The only minor complication associated with the BK ostomy closure was a small fistula that, due to its extraperitoneal nature, was successfully treated with conservative measures.

## CONCLUSION

Data from the present series allow us to affirm that the BK-type ostomy is a safe and efficient procedure that can be used primarily in selected cases as an alternative procedure to assess distal bowel function before a definitive transit reconstruction, in children with uncertain bowel motility.
